# Adaptive Estimation of Personalized Maximum Tolerated Dose in Cancer Phase I Clinical Trials Based on All Toxicities and Individual Genomic Profile

**DOI:** 10.1371/journal.pone.0170187

**Published:** 2017-01-26

**Authors:** Zhengjia Chen, Zheng Li, Run Zhuang, Ying Yuan, Michael Kutner, Taofeek Owonikoko, Walter J. Curran, Jeanne Kowalski

**Affiliations:** 1 Department of Biostatistics and Bioinformatics, Emory University, Atlanta, Georgia, United States of America; 2 Biostatistics and Bioinformatics Shared Resource, Winship Cancer Institute, Emory University, Atlanta, Georgia, United States of America; 3 Division of Biostatistics and Bioinformatics, Pennsylvania State University, Hershey, Pennsylvania, United States of America; 4 Department of Biostatistics, The University of Texas MD Anderson Cancer Center, Houston, Texas, United States of America; 5 Department of Hematology and Medical Oncology, Emory University, Atlanta, Georgia, United States of America; 6 Department of Radiation Oncology, Emory University, Atlanta, Georgia, United States of America; University of Michigan, UNITED STATES

## Abstract

**Background:**

Many biomarkers have been shown to be associated with the efficacy of cancer therapy. Estimation of personalized maximum tolerated doses (pMTDs) is a critical step toward personalized medicine, which aims to maximize the therapeutic effect of a treatment for individual patients. In this study, we have established a Bayesian adaptive Phase I design which can estimate pMTDs by utilizing patient biomarkers that can predict susceptibility to specific adverse events and response as covariates.

**Methods:**

Based on a cutting-edge cancer Phase I clinical trial design called escalation with overdose control using normalized equivalent toxicity score (EWOC-NETS), which fully utilizes all toxicities, we propose new models to incorporate patient biomarker information in the estimation of pMTDs for novel cancer therapeutic agents. The methodology is fully elaborated and the design operating characteristics are evaluated with extensive simulations.

**Results:**

Simulation studies demonstrate that the utilization of biomarkers in EWOC-NETS can estimate pMTDs while maintaining the original merits of this Phase I trial design, such as ethical constraint of overdose control and full utilization of all toxicity information, to improve the accuracy and efficiency of the pMTD estimation.

**Conclusions:**

Our novel cancer Phase I designs with inclusion of covariate(s) in the EWOC-NETS model are useful to estimate a personalized MTD and have substantial potential to improve the therapeutic effect of drug treatment.

## Introduction

It is common for a group of patients with the same cancer type to receive the same treatment. However, some patients will experience substantially better therapeutic effects than others, and some anticancer therapies may benefit only a subset of treated patients. Several reasons account for the heterogeneous therapeutic effect observed at the same dose level of the same drug. Patients have different genetic and environmental profiles, including demographic characteristics, concomitant diseases, concomitant drugs, biomarkers, SNP copy number, etc. [1,2,3]. Genetic and environmental factors interactively affect the therapeutic effect of a treatment intervention. Tumor heterogeneity is another significant reason for the heterogeneity of the toxicity and therapeutic effects of a drug. Tumors of a primary site in many cases represent a heterogeneous collection of diseases that differ with regard to the mutations that cause them and drive their invasion, thus are heterogeneous with regard to natural history and response to treatment. Personalized medicine has evolved recently as an advanced approach to achieve optimal medical effect in the context of a patient’s genetic, environmental, and tumor profiles [1,2,3,4,5].

The first critical step toward personalized medicine is the estimation of personalized maximum tolerated dose (MTD) in a Phase I clinical trial, which is the first trial of a new drug in humans after animal studies, with the main purpose to determine the MTD of a new drug under safe administration. In a Phase I clinical trial, there is substantial heterogeneity in dose limiting toxicity (DLT) response at the same dose level of the same drug among different patients because of different genetic and environmental profiles and tumor heterogeneity. Some known factors include the vulnerability to an exaggerated pharmaco-dynamic effect (potentially mediated by receptor differences), differences in genetic susceptibility (e.g. biomarker, G6PD deficiency), and drug–drug interactions [6,7]. Ignoring the potential heterogeneity may lead to serious bias in MTD estimation for different groups of patients [7,8], and as a result, the therapeutic effect is substantially decreased. Hence, in order to achieve the optimal therapeutic effect of a drug for every patient, estimating a personalized MTD offers greater potential than estimating an overall MTD across different patients [8].

The main goal of this study is to develop a practical and leading Phase I design that can facilitate the estimation of personalized MTD for the implementation of personalized medicine. Currently available Phase I designs can be classified as rule-based or model-based. Rule-based (or non-parametric) Phase I designs fail to estimate MTDs while adjusting for covariates due to their simple up and down algorithms. Therefore, a parametric or semi-parametric model-based design is desired so that covariates, especially genomic profiles, can be included into the dose response curve. Among several parametric Phase I designs available in the literature, Escalation With Overdose Control (EWOC), proposed by Babb, et al. [9], can control the probability of exceeding the MTD during the dose escalation phase and has been used in trials at Emory University, Fox Chase Cancer Center, Miami University, Novartis, and other institutions. EWOC can detect the true MTD with high accuracy, compared with traditional 3+3 designs. Nevertheless, EWOC only considers the worst toxicity event that a patient experiences. A binary outcome is used to denote whether the worst toxicity event that occurs has DLT status. Therefore, EWOC design is limited by its binary outcome when investigators want to consider not only DLT events but also non-DLT events. In a real clinical trial, patients often experience multiple toxicities with event grades varying from 0 (no toxicity of that type) to 5 (death). Some patients may experience multiple DLTs. In addition, the toxicity events are not equally severe. For example, a grade 4 renal toxicity of severe and possibly irreversible may be more severe than a grade 3 reversible toxicity of another type, such as grade 3 neutropenia. Moreover, some, but not all, low-grade non-DLT events that occur at a lower dose may be a sign of the occurrence of the same toxicity of a higher grade at a higher dose. Different toxicity events often occur together, such as fatigue and nausea/vomiting, myelosuppression and fever, etc. Considering both DLT and non-DLT events will allow the more accurate evaluation of the toxicity of a new drug [10,11,12,13].

We previously proposed a novel toxicity scoring system to overcome the above limitations by incorporating multiple toxicity responses as a quasi-continuous variable and thus, fully utilize all toxicity information [14]. The novel toxicity scoring system has two major components: Normalized Equivalent Toxicity Score (NETS) and Target NETS (TNETS). It has been successfully combined with the non-parametric Isotonic Design, to create a new semi-parametric design named ID-NETS. In Chen et al. [15], we further proposed to replace the binary outcome with NETS in the EWOC design so that the extended method (EWOC-NETS) can be used to evaluate the MTD by considering both DLT and non-DLT events. In this study, in order to estimate a personalized MTD, we propose to include baseline covariates that represent personal tumor, genetic, and environmental factors into the EWOC-NETS model. The selected baseline covariates must be biomarkers or classifiers with strong biological evidence that they may have some kind of linear relationship with the dose and drug effect, or that their categorical status can successfully predict the possibility of patient benefit from the agent. The varying MTD estimated in the novel method is the personalized MTD according to a patient’s tumor, genomic and environmental profile, instead of an overall MTD. In the remainder of this manuscript, we will describe in Section 2 details of the NETS system, EWOC-NETS, and the new model including the covariate. In Section 3, extensive simulations will be presented to examine the performance of EWOC-NETS after considering the covariate. In Section 4, we will discuss the limitations and applications of the new method.

## Methods

### 2.1. Major differences between EWOC and EWOC-NETS

EWOC-NETS was developed based on the framework of EWOC with two new concepts. The first is to fully utilize all toxicity information by replacing the binary outcome with the NETS (ranging from 0 to 1) which can be interpreted as the severity of a patient’s toxicity response. A quasi-likelihood function is further introduced to update the posterior marginal distribution of the MTD (*γ*). The second concept is that the MTD is re-defined as a dose corresponding to a pre-specified TNETS, *θ*, ranging from 0 to 1 instead of a Target Tolerated Level (TTL). The new definition satisfies

$$\theta =TNETS={ANETS}_{Dose=\gamma }$$ (1)

where ANETS is the Average NETS of the dose level. TNETS, θ, is an analog to the Target Toxicity Level (TTL) when using a binary DLT in a traditional clinical trial design and is determined by the Target Toxicity Profile, which is a more detailed toxicity response of a patient treated at MTD *γ*. EWOC-NETS assumes that the dose ANETS relationship follows a logistic model and NETS can also be interpreted as the observed fractional events ranging from 0 to 1 (see Fig 1 of Chen et al. [12]).

### 2.2. Quantitative measurement of toxicity response with NETS

Chen et al. [14] provide a detailed description of the method to derive NETS and determine TNETS for a clinical trial. Suppose in a trial with *K* patients, the *i*^th^ patient has a total of *N*_*i*_ toxicities. Toxicity events are assigned to adjusted grade according to 1 for grade 1 toxicity, 2 for grade 2 toxicity, 3 for grade 3 non-DLT, 4 for grade 4 non- DLT, 5 for grade 3 DLT, and 6 for grade 4 DLT (see Table 1 of Chen et al. [12]). Let *G*_*i*,*j*_ be the adjusted grade of the *j*^th^ toxicity (1 ≤ *j* ≤ *N*_*i*_) of the *i*^th^ patient. The maximum adjusted grade, *G*_*i*,*max*_, of the *i*^th^ patient can be obtained by Eq (2):

$$G_{i,max}=max(G_{i,j},j=1,\ldots ,J_{i})$$ (2)

Let *S*_*i*_ be the NETS of the *i*^th^ patient. If the *i*^th^ patient has no toxicity or only grade 1 toxicity (adjusted grade = 1), the *S*_*i*_ is arbitrarily assigned to 0 or 1/60, respectively. Otherwise, *S*_*i*_ can be derived by Eq (3).

$$S_{i}=\frac{1}{6}\left[G_{i,max}-1+\frac{exp(c+\beta *(\sum_{j=1}^{J_{i}}\frac{r_{i,j}G_{i,j}}{G_{i,max}}-1))}{1+\exp\left(c+\beta *(\sum_{j=1}^{J_{i}}\frac{r_{i,j}G_{i,j}}{G_{i,max}}-1)\right)}\right]$$ (3)

The parameter *r*_*i*,*j*_ ranging from 0 to 1 is a weight for the correlation of the *j*^th^ toxicity with other toxicities of the *i*^th^ patient. The weight decreases as the correlation increases. According to Chen et al. [14], the parameters *G*_*i*,*max*_ and *c* are suggested to be fixed at 6 and -2, respectively. The parameter β is the slope for the increasing rate of NETS controlling for toxicity events. The higher the value of β that is chosen, the more conservative the clinician is. β is recommended to be a value ranging from 0.1 to 0.5, such as 0.25.

### 2.3. Determination of TNETS

TNETS, θ, is an analog to the Target Toxicity Level (TTL) when using a binary DLT in a traditional clinical trial design. It is determined by the Target Toxicity Profile, which is a more detailed toxicity response of a patient treated at MTD. The calculation of TNETS, *θ*, is defined by

$$\theta =\sum_{l=0}^{6}m_{l}∙p_{l}$$ (4)

Here, *p*_*l*_ is the probability that the worst toxicity event is an adjusted grade *l* event and *m*_*l*_ is the corresponding mid-range NETS value when the toxicity with an adjusted grade of *l* is the “worst” toxicity for a patient with varying additional less severe toxicities in the target toxicity profile and the maximum adjusted grade for the patient is *l*. The range of the parameter *l* is from 0 to 6. In the above equation, *m*_0_ = 0, *m*_1_ = 0.092, *m*_2_ = 0.25, *m*_3_ = 0.417, *m*_4_ = 0.583, *m*_5_ = 0.75, and *m*_6_ = 0.917.

### 2.4. EWOC-NETS model including covariates

In an ideal situation, we need to fully consider all possible covariates and their interaction effects in order to estimate a personalized MTD. The full model of the dose and toxicity response relationship should include a constant, a vector of covariates including dosage, patients’ characteristics, other biomarkers, and the overall interaction terms as below.

S=F(Xβ+ε)

Where $S_{n\times 1}=\left[\begin{array}{c} S_{1}\\ S_{2}\\ .\\ .\\ .\\ S_{n} \end{array}\right],\;X_{n\times (m+2)}=\left[\begin{array}{ccccc} 1 & X_{1\times 1} & \cdots & X_{1\times m} & X_{1\times 1}\times X_{1\times 2}\times \cdots \times X_{1\times m}\\ \vdots & \vdots & \ddots & \vdots & \vdots \\ 1 & X_{n\times 1} & \cdots & X_{n\times m} & X_{n\times 1}\times X_{n\times 2}\times \cdots \times X_{n\times m} \end{array}\right]$,

$$\beta_{(m+2)\times 1}=\left[\begin{array}{c} \beta_{0}\\ \beta_{1}\\ .\\ .\\ .\\ \beta_{m}\\ \beta_{m+1} \end{array}\right],\;\varepsilon_{n\times 1}=\left[\begin{array}{c} \varepsilon_{1}\\ \varepsilon_{2}\\ .\\ .\\ .\\ \varepsilon_{n} \end{array}\right]$$

However, the small sample size of a Phase I trial makes it inadvisable to use an overly complicated model with too many covariates. Therefore, in the models proposed in this study, we limit to one covariate that is either discrete or continuous and have omitted the interaction term.

#### 2.4.1 Discrete covariate

Let *X*_min_ and *X*_max_ denote the minimum and maximum dose levels pre-specified by the clinician. Suppose a discrete covariate C with a value 1 means that the patient is in group A and a value 0 means that the patient is in group B. According to the pre-clinical trial, we know that group B generally has higher MTDs in males than in females. Let *γ*_max_ denote the MTD for group B and *γ*_0_ denote the MTD for group A. Therefore, the relationship

$$X_{min}\leq \gamma_{0}\leq \gamma_{max}\leq X_{max}$$ (5)

would be satisfied.

The dose assigned to the first patient is *X*_min_ and we shall select only dose levels between the interval of *X*_min_ and *X*_max._ Let *X*_*i*_ denote the dose assigned to the *i*^th^ patient, *i* = 1, 2, …, K, then *x*_1_ = *X*_min_ and *x*_i_ ∈ [*X*_min_,*X*_max_], ∀*i* = 1,…,*k*. We model the relationship between dose and ANETS by

$$\mu_{S_{i}|X_{i}}=F(\beta_{0}+\beta_{1}x_{i}+\delta c_{i})$$ (6)

where *F* is a pre-specified distribution function, called a tolerance distribution, and β_0_ and β_1_ are unknown. We assume that β_1_ > 0 and *δ* < 0 so that the ANETS monotonically increases when the dose level increases, adjusting for the covariate, *c*_*i*_. The MTD is the dose level, denoted by γ, such that the TNETS is θ. It follows from Eq 6 that

$$\theta =F(\beta_{0}+\beta_{1}\gamma_{max}+\delta )$$ (7)

where F is a logistic regression model here. We can further simplify Eq (7) to obtain Eq (8).

$$logit\;(\theta )=\beta_{0}+\beta_{1}\gamma_{max}+\delta$$ (8)

Let *ρ*_1_ denote the ANETS at the starting dose *x*_1_ = *X*_min_ for group A and *ρ*_2_ the ANETS at the starting dose *x*_1_ = *X*_min_ for group B.

$$logit\;(\rho_{2})=\beta_{0}+\beta_{1}X_{min}+\delta$$ (9)

$$logit\;(\rho_{1})=\beta_{0}+\beta_{1}X_{min}$$ (10)

Solving Eqs (8)–(10), we can re-parameterize the (β_0_, β_1_, *δ*) in terms of (*γ*_*max*_, *ρ*_1_, *ρ*_2_) by

$$\beta_{1}=\frac{logit(\theta )-logit(\rho_{2})}{\gamma_{max}-X_{min}}$$ (11)

$$\delta =logit(\rho_{2})-logit(\rho_{1})$$ (12)

$$\beta_{0}=logit(\rho_{1})-\frac{logit(\theta )-logit(\rho_{2})}{\gamma_{max}-X_{min}}X_{min}$$ (13)

(*γ*_*max*_, *ρ*_1_, *ρ*_2_) are interpretable to both clinicians and investigators. We can easily specify the non-informative uniform prior for (*γ*_*max*_, *ρ*_1_, *ρ*_2_) based on the pre-clinical trial. *ρ*_1_ and *ρ*_2_ are assumed to follow uniform distributions (0,*θ*). *γ*_*max*_ is assumed to follow uniform (*X*_*min*_, *X*_*max*_).

#### 2.4.2 Continuous covariate

The basic method to include a continuous covariate into the EWOC-NETS model is the same as the method to include a discrete covariate. However, the re-parameterization in Eq (13) is different. Let Z denote a continuous variable where the MTD increases when z increases, conditional on the same dose level. Then Eqs (8)–(10) should be changed by the new dose response relationship. Let *z*_*min*_ denote the smallest value of Z and *z*_*max*_ be the maximum of Z in the Phase I clinical trial. After considering age, the dose-response curve satisfies

$$logit\;(\theta )=\beta_{0}+\beta_{1}\gamma_{max}+\delta z_{max}$$ (14)

$$logit\;(\rho_{2})=\beta_{0}+\beta_{1}X_{min}+\delta z_{max}$$ (15)

$$logit\;(\rho_{1})=\beta_{0}+\beta_{1}X_{min}+\delta z_{min}.$$ (16)

A different re-parameterization for *δ* and *β*_0_ is derived by solving Eqs (14)–(16).

$$\delta =\frac{logit(\rho_{2})-logit(\rho_{1})}{z_{max}-z_{min}}$$ (17)

$$\beta_{0}=logit(\rho_{2})-\frac{logit(\theta )-logit(\rho_{2})}{\gamma_{max}-X_{min}}X_{min}\;\text{-}\;\frac{logit(\rho_{2})-logit(\rho_{1})}{z_{max}-z_{min}}z_{max}$$ (18)

### 2.5. Quasi-Bernoulli likelihood

Frequentist quasi-likelihood methods are designed to model overdispersion observed in binomial or Poisson data. When the “quasi” distributions belong to linear exponential families such as the binomial family, Quasi Maximum Likelihood Estimates (QMLEs) obtained by maximizing the quasi-Bernoulli likelihood function are strongly consistent [10,16,17,18]. Recently, the quasi-likelihood approach has been successfully combined with Bayesian generalized linear models [18] and the continual reassessment method (CRM) [10].

NETS can be viewed as fractional events. So, we assume that the variance structure of NETS (S) is ${\mu_{S_{i}|X}}_{i}(1-\mu_{S_{i}|X_{i}})$ conditional on dose level *X*_*i*_ for the i^th^ patient where *S*_*1*,*…*,_*S*_*N*_ are assumed to be mutually independent in a clinical trial with N patients. The quasi-Bernoulli likelihood can be applied to update the posterior distribution of (*γ*_*max*_, *ρ*_1_, *ρ*_2_) under these assumptions.

If we want to include a continuous variable in our model, the data after observation of *k* patients can be expressed as *D*_*k*_ = {(*x*_*i*_, *z*_*i*_, *s*_*i*_), *i* = 1, 2, …, *k*}. *D*_*k*_ would include the dose assigned (*x*_*i*_), the NETS observed (*s*_*i*_), and the continuous covariate (*z*_*i*_) of each previously treated patient. The quasi-Bernoulli likelihood of (*γ*_*max*_, *ρ*_1_, *ρ*_2_) given *D*_*k*_ is

$$L(\gamma_{max},\rho_{1},\rho_{2}|D_{k})=\prod_{i=1}^{N}\mu_{S_{i}|X_{i}}^{s_{i}}{\left(1-\mu_{S_{i}|X_{i}}\right)}^{{1-s}_{i}}$$ (19)

Obviously, the true distribution in NETS is unknown so we try to make inferences based on the known variance structure and the corresponding quasi-likelihood. According to the quasi-Bayesian theory, the quasi-likelihood function can be interpreted as the “limited information likelihood” and it is the best approximation of the true likelihood. After plugging in the corresponding dose ANETS curve, the likelihood function can be written as

$$L(\gamma_{max},\rho_{1},\rho_{2}|D_{N})=\prod_{i=1}^{N}{{F(\beta_{0}+\beta_{1}x_{i}+\delta z_{i})}^{s_{i}}(1-F(\beta_{0}+\beta_{1}x_{i}+\delta z_{i}))}^{{1-s}_{i}}$$ (20)

### 2.6. Overdose control in EWOC-NETS

The traditional Bayesian decision theory makes inferences of the posterior mean, median or mode so that the corresponding expected loss function can be minimized. In order to control the overdose rate, we do not choose the posterior median of EWOC-NETS as an estimator of MTD at the beginning of the clinical trial. Instead, the *α*^th^ percentile of the posterior marginal distribution is chosen. As a result, the marginal posterior overdosing probability is equal to α for the next patient. α was referred to as the feasibility bound by Babb, et al [9]. Here, we generally present how to extend overdose control after including a continuous covariate Z.

The posterior distribution after the observation of *k* patients can be derived by

$$p_{k}(\gamma_{max},\rho_{1},\rho_{2}|D_{k})\propto L(\gamma_{max},\rho_{1},\rho_{2})*p(\gamma_{max},\rho_{1},\rho_{2})\propto L(\gamma_{max},\rho_{1},\rho_{2})$$ (21)

where *p*(*γ*_*max*_, *ρ*_1_, *ρ*_2_) is the joint prior of the new parameter (*γ*_*max*_, *ρ*_1_, *ρ*_2_). We assume that the priors for *γ*_*max*_, *ρ*_1_, *ρ*_2_ are mutually independent and follow uniform distributions. The posterior joint distribution of (*γ*_*max*_, *ρ*_1_, *ρ*_2_) is dominated by the observed data.

The MTD of the (k+1)th patient adjusting for *z*_*k+1*_ can be re-parameterized as $\gamma_{z_{k+1}}=\gamma_{max}+\frac{\delta }{\beta_{1}}(z_{max}-z_{k+1})$. The posterior distribution of $\gamma_{z_{k+1}}$ can be updated by the joint posterior distribution of (*γ*_*max*_, *ρ*_1_, *ρ*_2_). We denote the cumulative density function (CDF) for $\gamma_{z_{k+1}}$ as $\pi_{k,z_{k+1}}(∙|D_{k}).\;\pi_{k,z_{k+1}}(∙|D_{k})$. It is not only related to the cumulative data *D*_*k*,_ but also related to the next patient’s covariate *z*_*k+1*_. In order to ensure the ethical constraint of overdose control, the selected dose *x*_*k*+1_ for the new patient must satisfy equation:

$$\pi_{k,Z_{k+1}}(x_{k+1}|D_{k})=\alpha .$$ (22)

At the end of the trial, the personalized MTD is the posterior median of the corresponding posterior marginal distribution adjusting for the covariate.

During the implementation of Bayesian procedures, the posterior distributions are estimated using the Markov Chain Monte Carlo (MCMC) method. The MCMC method has been widely used in Bayesian frameworks to sample posterior distributions with high dimensional parameters [19]. The Metropolis–Hastings algorithm is used to obtain a sequence of random samples). In the EWOC-NETS design, the burn-in period is 1000 iterations with another 1000 iterations conducted to sample the posterior distribution. Tighiouart et al. [20] reported a successful example of using MCMC to study a large class of prior distributions in EWOC.

### 2.7. Additional advantages of EWOC-NETS

Simulation studies and their application to real clinical trial data demonstrate that EWOC-NETS maintains the advantages of EWOC. In addition, it provides new advantages: 1) treats toxicity response as a quasi-continuous variable; 2) improves the MTD accuracy by differentiating beyond the DLT; 3) increases trial efficiency by fully utilizing all toxicities [15].

## Results

### 3.1 Models incorporating patient characteristics and biomarkers

In this manuscript, we use EWOC-NETS as a framework to incorporate patient characteristics and biomarkers. The original EWOC-NETS without considering any covariates is used as a baseline model for comparison. Therefore, a total of 3 models is considered: 1) Model 1 is the original EWOC-NETS model not including any covariates (baseline model); 2) Model 2 is the EWOC-NETS model considering only a binary covariate C (0 or 1); 3) Model 3 is the EWOC-NETS model considering only a continuous covariate Z (0 ~ 1). The logistic model of the relationship between NETS and dose *x* for each of the 3 models is summarized below:

$$\begin{array}{l} Model\;1:\;logit\;\mu_{S_{i}|X_{i}}=\beta_{0}+\beta_{1}X_{i}\\ Model\;2:\;logit\;\mu_{S_{i}|X_{i},C_{i}}=\beta_{0}+\beta_{1}X_{i}+\beta_{2}C_{i}\\ Model\;3:\;logit\;\mu_{S_{i}|X_{i},Z_{i}}=\beta_{0}+\beta_{1}X_{i}+\beta_{2}Z_{i} \end{array}$$

The corresponding parameters are re-parameterized as (*γ*_*max*_, *ρ*_1_, *ρ*_2_) as mentioned in Section 2. In order to evaluate the additional advantages in the performance of EWOC-NETS after considering covariates, model 2 and model 3 are compared to the baseline model 1 using bias, standard error (SE) and mean square error (MSE), respectively, under different scenarios.

### 3.2 Simulation settings

In order to compare the performance of the extended methods and the original method, models 2 and 3 are evaluated mainly under four scenarios each. The *γ*_*max*_ is defined as the true MTD for the group with C = 1 and *γ*_0_ is defined as the true MTD for the group with C = 0 when the covariate is discrete or continuous. The simulation set up under the 8 scenarios (S1 to S8) is summarized in Table 1. The first 4 scenarios (S1 to S4) are for discrete covariates and the other 4 scenarios (S5 to S8) are for continuous covariates. The covariates considered actually have true effects on MTDs under S1, S2, S3 for discrete covariates and under S5, S6, S7 for continuous covariates. The *γ*_*max*_ is set to 0.5 and *γ*_0_ values are, respectively, 0.27, 0.38, 0.44 under scenario S1, S2 and S3 for model 2. The same *γ*_0_ values are assigned under S5, S6, and S7 for model 3. There are no true effects on MTD under S4 for discrete covariates and under S8 for continuous covariates, respectively, so that *γ*_*max*_ and *γ*_0_ are both set to 0.5 under both scenarios. In order to obtain comparable results, the dose *X* is standardized within the range from 0 to 1; the continuous covariate Z is also standardized within the range from 0 to 1; and the binary covariate C is either 0 or 1. The ANETS is generated by the true tolerated function. The NETS is generated by a truncated normal distribution with the mean, ANETS, and the assumed variance structure. Under S1, S2, S3 and S4, we enroll 15 cohorts with covariates of value 1 (C = 1) and 15 cohorts with covariate of value 0 (C = 0). The group with C = 1 is treated first because we assume that their MTD is higher and it is safer to treat them first. Under S5 to S8, the continuous covariate values are generated from a uniform distribution (0, 1).

**Table 1:** Simulation set-up in each scenario and simulation results from model 1 (EWOC-NETS considering no covariate) under different scenarios.

Simulation set-up in each scenario			Estimation from Model 1 considering no covariate					
Scenario	True value of *γ*_*max*_	True value of *γ*_0_	MTD Mean	SE	*γ*_*max*_		*γ*_0_	
					Bias	MSE	Bias	MSE
S1	0.5	0.27	0.356	0.020	-0.144	0.021	0.079	0.007
S2	0.5	0.38	0.436	0.027	-0.064	0.005	0.068	0.005
S3	0.5	0.44	0.493	0.034	-0.007	0.001	0.055	0.004
S4	0.5	0.5	0.542	0.040	0.042	0.003	0.042	0.003
S5	0.5	0.27	0.407	0.034	-0.093	0.010	0.130	0.018
S6	0.5	0.38	0.463	0.035	-0.037	0.003	0.095	0.010
S7	0.5	0.44	0.506	0.037	0.006	0.001	0.069	0.006
S8	0.5	0.5	0.543	0.041	0.043	0.004	0.043	0.004

The comparisons of the performances of the different models are based on: 1) whether the estimated MTD is a personalized MTD; 2) the amount of bias of the final estimation of ${\hat{\gamma }}_{max}$, defined as $\sum_{i=1}^{n}({\hat{\gamma }}_{max}-\gamma_{max})/n$, where n is the total number of simulations; 3) the mean square error (MSE) of the final estimation of ${\hat{\gamma }}_{max}$ where a smaller MSE yields better performance of the models; and 4) the standard error (SE) of the estimator ${\hat{\gamma }}_{max}$ where the lower the standard error the more stable is the estimator.

In each simulated trial, the TNETS level is set to 0.476, which is calculated based on a target toxicity level of 33% DLT with a target toxicity profile consisting of equal probability for each non-DLT or DLT toxicity, respectively. The sample size is set to 30 in each simulated trial. The feasibility bound, α, is set to start at 0.25 for every model. α is increased with an increment unit of 0.05 when we assign the dose to the next cohort. The maximum of the feasibility bound is 0.5. The trial starts with the lowest dose level and the recommended dose level for the next cohort is the α^th^ percentile of the posterior marginal distribution of the MTD adjusting for its covariate. Each scenario is simulated 1,000 times. We define the limiting NETS status (LNETS) as 1 if the observed NETS exceeds 0.476, and 0 otherwise. Meanwhile, since we choose a dose level based on a continuous scale, we assume a tolerance, 0.05, for both overdosing and limiting NETS status.

*γ*_*max*_, *ρ*_1_ and *ρ*_2_ are respectively assumed to follow mutually independent priors: uniform distribution (0, 1), uniform distribution (0, 0.476), and uniform distribution (0, 0.476), respectively. The posterior sample of (*γ*_*max*_, *ρ*_1_, *ρ*_2_) is directly sampled by the Metropolis-Hastings algorithm implemented by JAGS, an efficient software for sampling the MCMC chain. The burn-in period is 1000 iterations. We use an additional 1000 iterations as the posterior sample of (*γ*_*max*_, *ρ*_1_, *ρ*_2_). Trace plots and histograms are used to diagnose whether the MCMC chain has converged. The histogram and trace plot for the parameter of primary interest, *γ*_*max*_ have shown that after 2000 iterations, the MCMC chain is stable and becomes a unimodal curve so that the posterior sample can provide more evidence to infer the parameter of interest *γ*_*max*_ by the posterior median.

### 3.3 Inclusion of a discrete covariate

Comparison of the accuracy of the estimated parameters between model 1 and 2 is summarized in Tables 1 and 2. The advantage of model 2 is obvious when there is a huge difference between two groups (C = 0 and C = 1) in S1. Model 2 successfully detects a different MTD for the two groups when the true MTD for the C = 0 group increases from 0.27 to 0.5. In contrast, model 1 always estimates only one MTD for both groups although these two groups should have two different MTDs (Table 1 and 2). The bias for the MTD estimation using model 2 is the smallest in S1, respectively, 0.058 and -0.013 (Table 2). The standard error and MSE are also the smallest in this scenario (Table 2). The bias, standard error and MSE increase when the difference between the MTD of the two groups decreases from S1 to S4 (Table 2). The results show that the MTD estimation for the two groups using model 2 will be more precise when the covariate effect on ANETS is larger (Table 2). Under the worst scenario S4, model 2 estimates *γ*_*max*_ as 0.6 and *γ*_0_ as 0.487, both of which are close to their true values (0.5), suggesting the robustness of model 2 when the covariate considered has no true effect. On the other hand, the bias, standard error and MSE for model 1, which only estimates a mean MTD for two groups, tends to decrease as the covariate effect on the toxicity outcome in terms of ANETS decreases from S1 to S4 (Table 1). Therefore, the MTD estimation for model 1 will be more precise when the covariate effect on ANETS diminishes (Table 1). More patients are required when the covariate effect on ANETS is small.

**Table 2:** Simulation results from model 2 (EWOC-NETS considering a discrete covariate) and 3 (EWOC-NETS considering a continuous covariate) under different scenarios.

Models Consider a Covariate	Scenario	*γ*_*max*_				*γ*_0_			
		Mean	Bias	SE	MSE	Mean	Bias	SE	MSE
Model 2 (Discrete covariate)	S1	0.558	0.058	0.041	0.005	0.263	-0.013	0.034	0.001
	S2	0.584	0.084	0.046	0.009	0.355	-0.014	0.044	0.002
	S3	0.593	0.093	0.050	0.011	0.426	-0.011	0.057	0.003
	S4	0.600	0.100	0.051	0.013	0.487	-0.013	0.064	0.004
Model 3 (Continuous covariate)	S5	0.532	0.032	0.045	0.009	0.268	-0.009	0.044	0.006
	S6	0.562	0.062	0.050	0.019	0.349	-0.019	0.056	0.011
	S7	0.580	0.080	0.049	0.026	0.408	-0.029	0.066	0.016
	S8	0.593	0.093	0.052	0.034	0.468	-0.032	0.077	0.021

**Fig 1:**
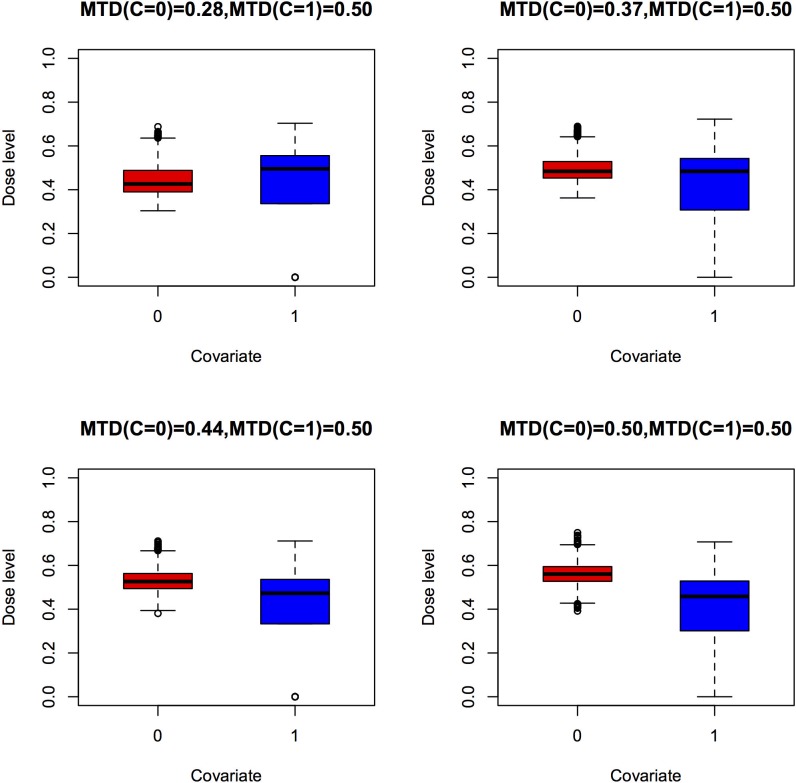
Patient distribution box plots for model 1, which does not consider a discrete covariate.

**Fig 2:**
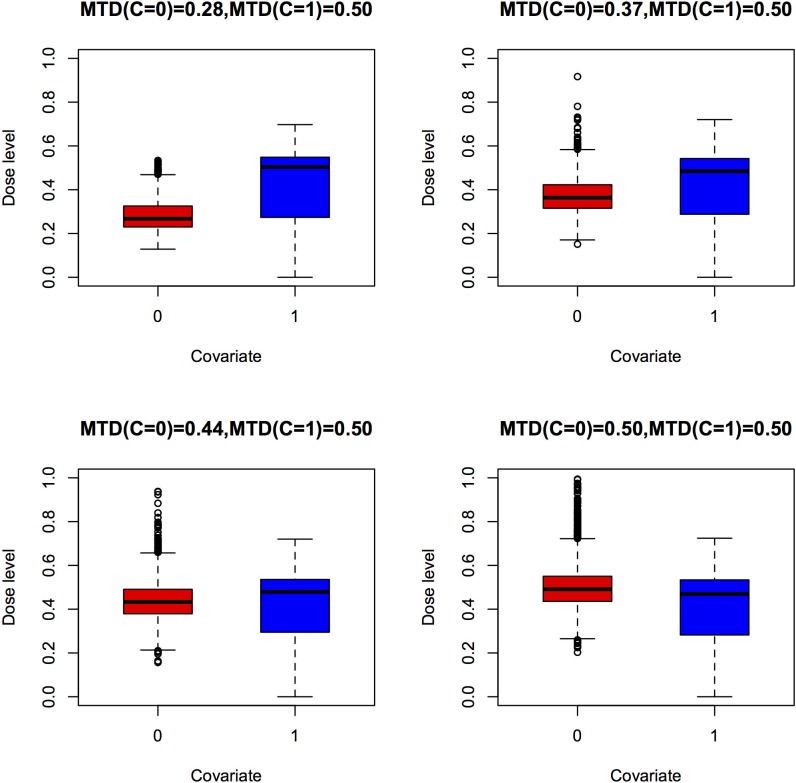
Patient distribution box plots for model 2, which considers a discrete covariate.

Compared with model 2, MTD estimation is a pooled estimate of the two groups of patients. As a result, the bias of the pooled estimator for the two groups is larger when the covariate effect on ANETS increases. Because of the small sample size in each group in the trial, the standard error of the MTD estimation is larger in model 2. The patient distributions among different dose levels under model 1 and model 2 are shown by the box plots in Fig 1 and Fig 2, respectively. Patients in different groups are treated at dose levels concentrated around their personalized MTD under model 2 (Fig 2). By contrast, patients are concentrated around the pooled estimation of MTD under model 1 (Fig 1). Since a dose level near the MTD is more effective and safer, the therapeutic effect for patients is better under model 2 than under model 1. The comparison of operating characteristics between model 1 and model 2 in terms of the overdosing rate, as measured by MTD+ percentage and LNETS percentage, is summarized in Table 3. The rates for patients being treated at dose levels higher than the MTDs (MTD+%) in all scenarios are smaller under model 2 than under model 1. Similarly, the rates for dosing above the limiting NETS status (LNETS+%) are lower under model 2 than model 1 among all scenarios. The difference between the overdosing rates of model 2 and model 1 increases as the difference in MTDs of the two groups increases when stratified by a discrete covariate. For example, the LNETS+ rate in scenario S1 is decreased by 22% under model 2 (39%) compared to the rate under model 1 (61.6%). The inclusion of the discrete covariate not only makes the final MTD recommendation more precise but also increases the therapeutic effect, and is also more ethical for patients.

**Table 3:** Comparison of overdosing rates in 1000 simulations.

**Scenario**	**MTD+ (%)**		**LNETS (%)**	
	**Model 2**	**Model 1**	**Model 2**	**Model 1**
S1	24.4	63.8	39.0	61. 6
S2	24.1	58.4	36.9	52.5
S3	23.5	49.8	35.7	46.3
S4	22.8	38.0	35.5	41.1
	**Model 3**	**Model 1**	**Model 3**	**Model 1**
S5	30	37	43	48
S6	33	40	41	46
S7	35	40	40	44
S8	35	39	38	41

### 3.4 Inclusion of a continuous covariate

Comparison of the accuracy of the estimated parameters between model 3 and model 1 under the 4 scenarios (S5 to S8) is summarized in Tables 1 and 2. Under the ideal scenarios (S5, S6, and S7) in which the continuous covariate considered has a true effect on the MTD, model 3 can estimate a personalized MTD depending on the value of the continuous covariate of a patient (Z). The baseline EWOC-NETS without considering any covariate (model 1) fails to estimate a personalized MTD as it can only estimate the marginal MTD for all patients with different covariate values. The bias, standard error and MSE of the MTD estimation become smaller under model 3 when the continuous covariate effect on ANETS is larger (Table 2). However, the bias, standard error, and MSE for model 1 decrease when the true effect of the continuous covariate decreases from S5 to S8 (Table 2). Under the worst scenario S8, model 3 estimates *γ*_*max*_ as 0.593 and *γ*_0_ as 0.468, both of which are close to their true values (0.5) (Table 2). This demonstrates the robustness of model 3 under all scenarios.

Comparisons of the overdosing rates between model 3 and model 1 are summarized in Table 3. Under scenario S5, consideration of a continuous covariate in EWOC-NETS (model 3) can reduce the MTD+ rate from 37% to 30% and the LNETS rate from 48% to 43%, resulting in better therapeutic effect for the participating patients in the Phase I trial (Table 3). When the considered continuous covariate actually has no true effect in MTD (S8), the MTD+ rate and LNETS rate of model 3 are still lower than the corresponding rates under model 1. This suggests that we will lose little when we consider a continuous covariate without a true effect on the MTD.

### 3.5 Sample size and number of covariates

From Sections 3.3 and 3.4, we can see that covariates should be included in the model when they have a true effect and that considering non-effective covariates has little loss under the worst scenario. The sample size of each covariate value is important when we consider the inclusion of the covariate because a small sample size of each covariate value makes the posterior estimation unstable in 1000 simulations. In order to estimate the MTD more precisely, more patients with the same covariate value are needed in a real clinical trial.

### 3.6 Applications

The above simulations are hypothetical/computer-generated in order to demonstrate the operating characteristics of the novel designs. The designs could be applied to available Phase I data/covariates to confirm their utility. For example, using the designs, three Phase I clinical trials have been designed to incorporate patient characteristics and biomarkers to estimate pMTDs for head and neck cancer patients at the Winship Cancer Institute of Emory University: 1) To estimate pMTDs for panitumumab according to each patient’s binary status of human papilloma virus (HPV) (positive vs negative); 2) To estimate pMTDs for folate-dextran-paclitaxel (FDT) according to each patient’s folate receptor level (continuous); 3) To estimate pMTDs for luteolin according to each patient’s specific type of head and neck cancer (categorical).

## Discussion

The concept of personalized medicine was introduced as early as the 1970s. Byar et al. originally proposed to select optimal treatment in clinical trials using covariate information [21]. Substantial advances have been made in this century due to progress in modern sequencing technologies, the development of new therapeutics, and the significant contribution of clinical trial methodologies. Personalized medicine has become a crucial component of contemporary cancer medicine. Examples of successful personalized medicines include several that target the epidermal growth factor receptor (EGFR). Imatinib (Gleevec), a tyrosine-kinase inhibitor of EGFR, was initially invented in the late 1990s by a biochemist, Nicholas Lyndon, and then approved by the FDA in 2001 for the treatment of multiple cancers, most notably Philadelphia chromosome-positive (Ph^+^) chronic myelogenous leukemia (CML)[22]. Imatinib has benefitted thousands of patients with CML and gastrointestinal stromal tumors (GIST). Cetuximab, an EGFR antibody, has been approved by the FDA for the treatment of colon cancer with wild-type KRAS, but not those with a KRAS mutation [23], and for the treatment of certain stages of head and neck cancer. Panitumumab, a fully human monoclonal antibody specific to the EGFR, has been approved by the FDA for the treatment of EGFR-expressing metastatic colorectal cancer with disease progression [24]. In 2012, the FDA approved a real time PCR companion diagnostic test for KRAS, Therascreen KRAS test, which is the first genetic test to guide the treatment of cancer. Recent advances in biotechnology have resulted in a shift toward molecularly targeted anticancer agents, which are likely to benefit only a subset of the patients with a given cancer. Due to the molecular heterogeneity of most human cancers, only a subset of treated patients benefit from a given therapy. This is particularly relevant for the new generation of anticancer agents that target specific molecular pathways. When biomarkers to identify patients who are likely to benefit from a given therapy are available, targeted clinical trials that restrict eligibility to sensitive patients can be conducted. The identification of the appropriate “sensitive” population requires definitive testing of specific biomarkers. Ideally, such diagnostic tests should be developed and validated before designing the trial. New biotechnologies such as microarrays can be used as powerful tools to measure biomarkers or a genetic signature and identify patients that are most likely to benefit from anticancer therapies. For example, Derin et al. reported that a lower level of MAPK expression is associated with anthracycline resistance and decreased survival in patients with hormone receptor negative breast cancer [25]. Fan et al. summarized the concordance among gene expression-based predictors for breast cancer [26]. Rapid advances in biotechnology have made it possible to obtain quantitative information regarding biomarkers and differentiate patient subsets according to sensitivity to agents. For example, EGFR is a target for molecularly targeted agents in lung cancer therapy. EGFR expression is first measured immunohistochemically on a continuous or graded scale, and is later used to categorize patients into several distinct categories for clinical management or dichotomize with various cut points, such as in the DAKO kit [27].

Personalized medicine has thus become a significant and valuable new approach in the field of medicine and the estimation of a personalized MTD according to a patient’s environmental and genetic profile is a critical step toward personalizing their treatment. The goal of this study is to propose an extended Phase I design which can fully utilize all toxicity information and patient’s characteristics to estimate personalized MTDs for precision medicine. Through simulation studies, we demonstrate the advantages and potential loss of estimating the conditional MTD given covariates using the EWOC-NETS design proposed by Chen et al. [15]. From the simulations, we can conclude that the extended EWOC-NETS incorporating patient characteristics and biomarkers can: 1) estimate a personalized MTD; 2) reduce the probability of patients being overdosed; 3) increase the accuracy of the treated dose, thus improving the therapeutic effect; and 4) have little loss when covariates being considered have no effect.

Some other methodologies have been proposed to estimate personalized MTDs in Phase I clinical trials. For example, O’Quigley et al. proposed to estimate different MTDs for two groups or ordinal groups of patients stratified by binary or ordinal covariates based on the CRM design [28,29]. Based on EWOC, Babb et al. proposed to include a continuous covariate in the dose toxicity relationship model and estimate patient specific dosing in a cancer phase I clinical trial [30]. Tighiouart et al. further used the EWOC design to incorporate patient’s dichotomous characteristics and estimate patient specific MTD [31]. Our method is based on EWOC-NETS which is an extension of EWOC that uses NETS instead of the probability of DLT. The comparison of performance between EWOC and EWOC-NETS has been elaborated in previous publications [14,15,32,33]. Our results based on EWOC-NETS are consistent with the findings of O’Quigley et al. [28,29], Babb et al [30], and Tighiouart et al [31]. Besides the Phase I clinical trial methodologies developed to determine personalized MTD, other novel approaches have been proposed to use biomarkers or genomic signatures for personalized medicine in Phase II/III clinical trials in oncology and other diseases [1,2,3,4,5]. For example, Freidlin and Simon proposed an adaptive signature design to generate and prospectively test a gene expression signature for sensitive patients [5]. Jiang et al. further developed a biomarker-adaptive threshold design for evaluating treatment with a possible biomarker-defined subset effect [1]. Freidlin et al. recently extended their adaptive signature design and developed a cross validated adaptive signature design which has considerable improvement in performance [2].

Our extended EWOC-NETS design can be applied to estimate the personalized MTD of a new agent according to the status of specific biomarkers of the targeted cancer. When planning a Phase I trial, we need to decide whether or not to include patient covariates and which covariates to include in the MTD estimation during the trial. Some well-known biomarkers and their related diseases are summarized in Table 4. Each of these biomarkers as well as other patient characteristics can be treated as binary or continuous covariates and incorporated into the corresponding models. The Phase I clinical trial is typically a small study with a small sample size and as such we cannot attempt to estimate too many parameters. Therefore, when multiple biomarkers need to be considered, we can estimate their combined effect as a single score by using an additive linear model and incorporating the score into the model as a continuous or binary variable. We should not consider any covariates that are known to have no effect on the MTD. From a statistical point of view, we stand to lose little in terms of the accuracy of the MTD and therapeutic effect for participants when covariates taken into account in the model are actually not predictive of the severity of patients’ toxicity response. However, it is a substantial monetary cost to quantify covariates, especially when patients need to be genotyped and certain biomarker expressions need to be determined. Therefore, careful consideration must be given to the balance of these pros and cons.

**Table 4:** Some well-known biomarkers and associated diseases.

Cancer/disease type	Biomarker
Non-small cell lung cancer	HER2, EGFR, KRAS, UGT1A1, etc.
Head and neck cancer	EGF, VEGF, Cox2, G-CSF, GM-CSF, ErbB2, EGFR, etc.
Breast cancer	BRCA1/2, Her-2/neu receptor
Colorectal cancer	EGFR, KRAS, ERCC, RRM1, etc.
Acute myeloid leukemia	Cd33, FLT3, inv16
Non-Hodgkin’s lymphoma	CD20, MALT
HIV	HLA-B*5701, CCR5

Subsequent Phase II and III trials should further test the efficacy of personalized MTD among biomarker-stratified patients instead of testing an average effect among an unselected population. In an enrichment design, all patients will be homogeneous regarding a specific binary biomarker, so that the dose recommended to all participants will be the personalized MTD according to the status of the biomarker. In a biomarker-stratified design, patients of each stratum will be treated with a specific pMTD according to the level of the categorical biomarker the patient has in the stratum. In more complicated settings, where no natural cut-point of the biomarker is known in advance, or there are multiple biomarkers and high dimensional genomic tumor characterization, patients will be treated with pMTD according to the value of a continuous biomarker or a composite score summarizing their overall effect.

There are also some limitations in the real practice of the design. For example, the required genomic or demographic data from incoming patients may need to be retrieved from different sources and be integrated together for pMTD determination. Different data are not always available immediately and sometimes may be inconsistent. Therefore, reliability, validation, and standardization of data from different sources are critical during the data processing, storage, analysis, and interpretation to make real-time personalized assessments. Most previous studies have been conducted based on an individual study level. Patient privacy during the sharing of data is another consideration. Fortunately, increasing computation capability, rapidly expanding databases, and powerful safe networks enable the immediate retrieval and integration of bioinformatics and clinical data to deliver real-time information for pMTD estimation and generate a highly personalized outcome while protecting patient privacy. Further database development is highly recommended for the estimation of pMTD and implementation of personalized medicine, but the application of our Phase I design for pMTD should not be limited by the availability of related databases. Biomarkers have played significant roles in the adaptive randomization, group stratification, and patient enrichment of Phase II and III clinical trials. The pMTD estimated from a Phase I trial should be utilized to treat different groups of patients with personalized doses according to their genomic and clinical characteristics, thus optimizing the efficacy of precision medicine and maximizing therapeutic effects. Given the potential benefits of personalized therapy, the estimation of pMTD as the first step toward this goal should be pursued whenever at least some genomic and clinical data are available.

In summary, our study has shown that the inclusion of covariate(s) in the EWOC-NETS model is useful to estimate a personalized MTD and has substantial potential to improve the therapeutic effect of drug treatment.
